# Supplementary figures and images for: The drift diffusion model as the choice rule in inter-temporal and risky choice: A case study in medial orbitofrontal cortex lesion patients and controls
Source: PLoS Comput Biol. 2020 Apr 20;16(4):e1007615. doi: 10.1371/journal.pcbi.1007615 (PMC7192518; doi:10.1371/journal.pcbi.1007615)

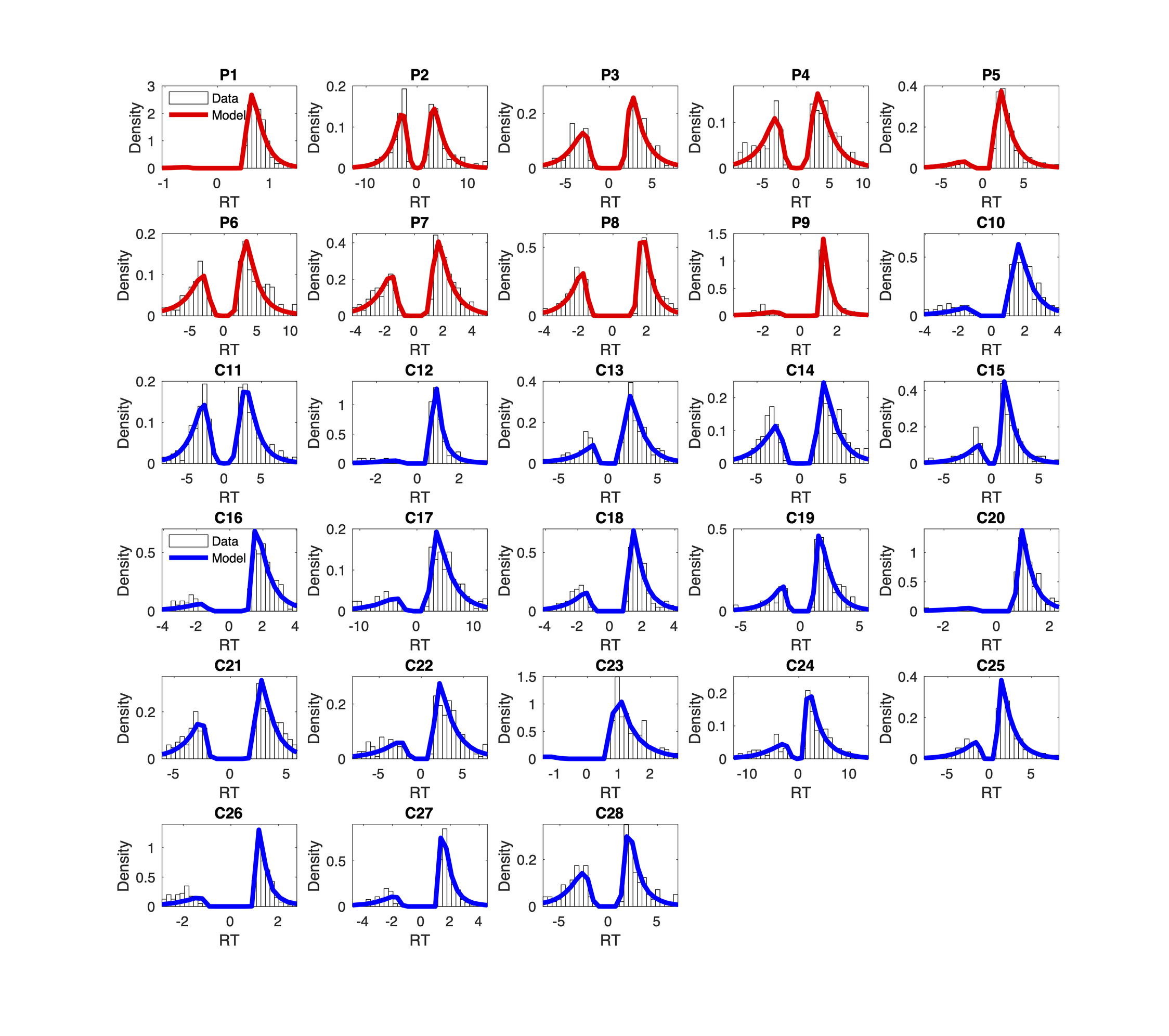

Supplement: S1 Fig — Histograms depict the observed RT distributions for each participant. The solid lines are smoothed histograms of the model predicted RT distributions from 1000 individual subject data sets simulated from the posterior distribution of the best-fitting hierarchical model. RTs for smaller-sooner choices are plotted as negative, whereas RTs for larger-later choices are plotted as positive. The x-axes are adjusted to cover the range of observed RTs for each participant. (TIF) [file pcbi.1007615.s001.tif]

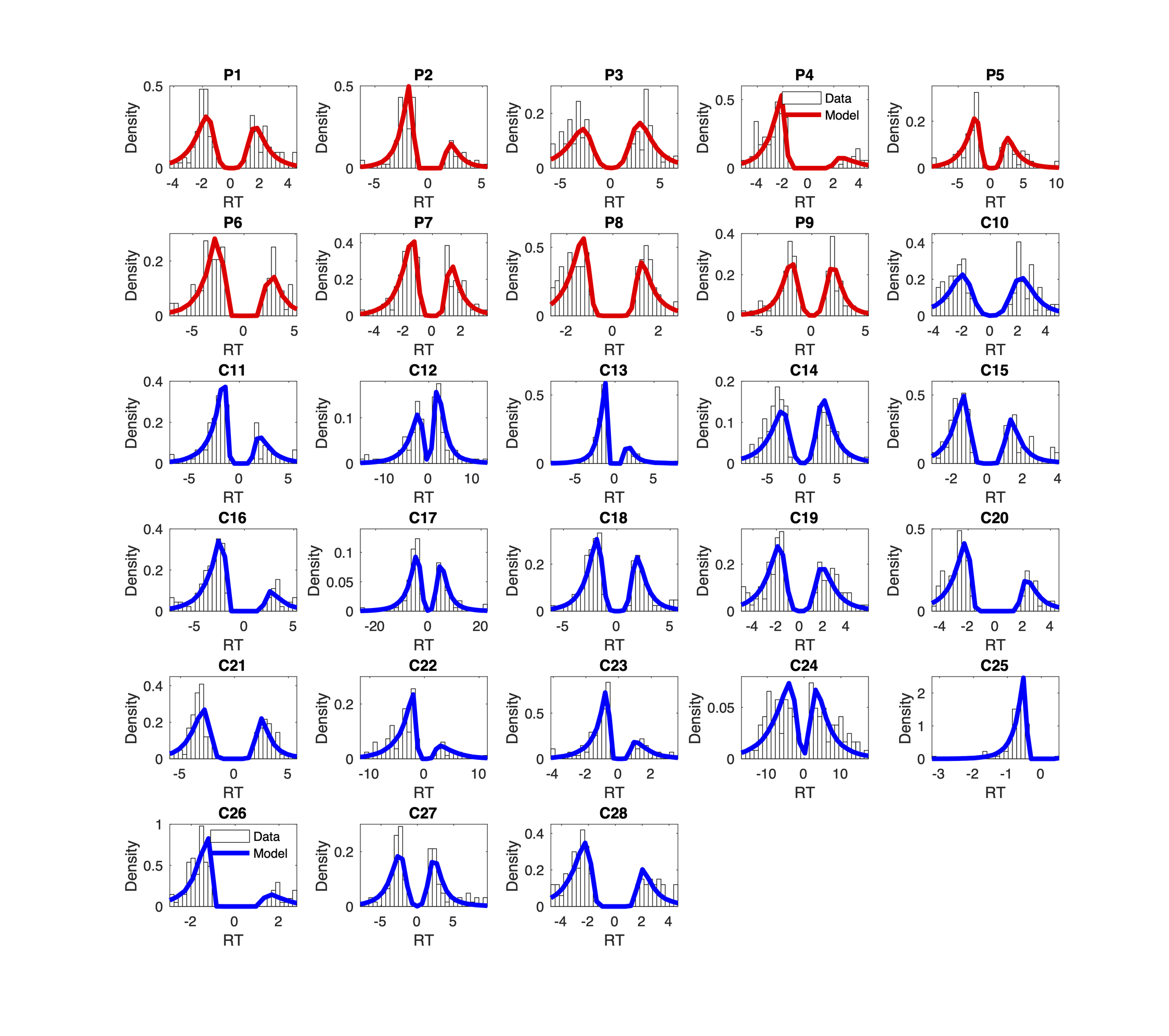

Supplement: S2 Fig — Histograms depict the observed RT distributions for each participant. The solid lines are smoothed histograms of the model predicted RT distributions from 1000 individual subject data sets simulated from the posterior distribution of the best-fitting hierarchical model. RTs for choices of the safe option are plotted as negative, whereas RTs for risky choices are plotted as positive. The x-axes are adjusted to cover the range of observed RTs for each participant. (TIF) [file pcbi.1007615.s002.tif]

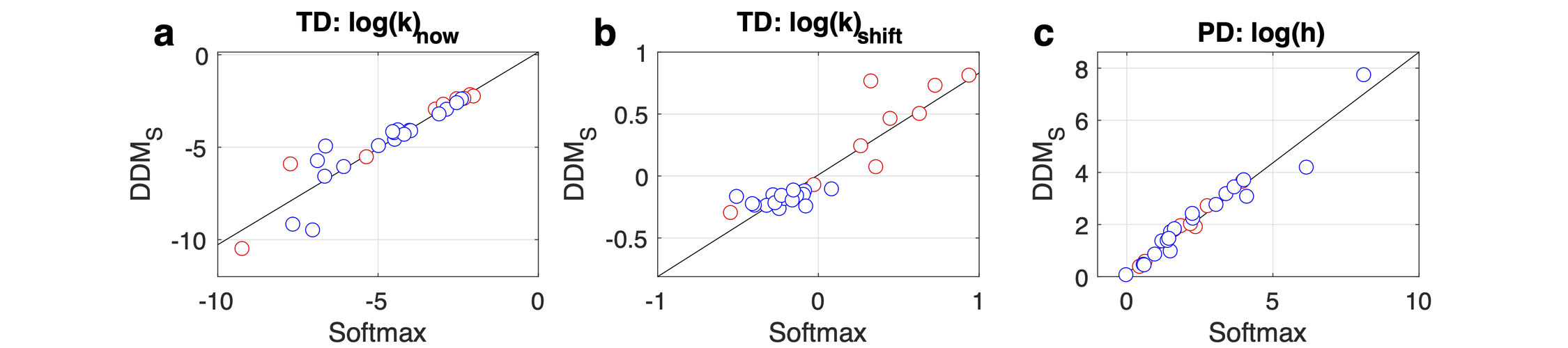

Supplement: S3 Fig — Scatter plots (controls: blue, mOFC patients: red) show model parameters estimated via a standard softmax choice rule (x-axis) vs. parameters estimated via a drift diffusion model choice rule with non-linear drift rate scaling (DDMS, y-axis). a) Temporal discounting log(discount rate) for now trials. b) Shift in log(k) between now and not now trials). c) Probability discounting log(discount rate). (TIF) [file pcbi.1007615.s003.tif]

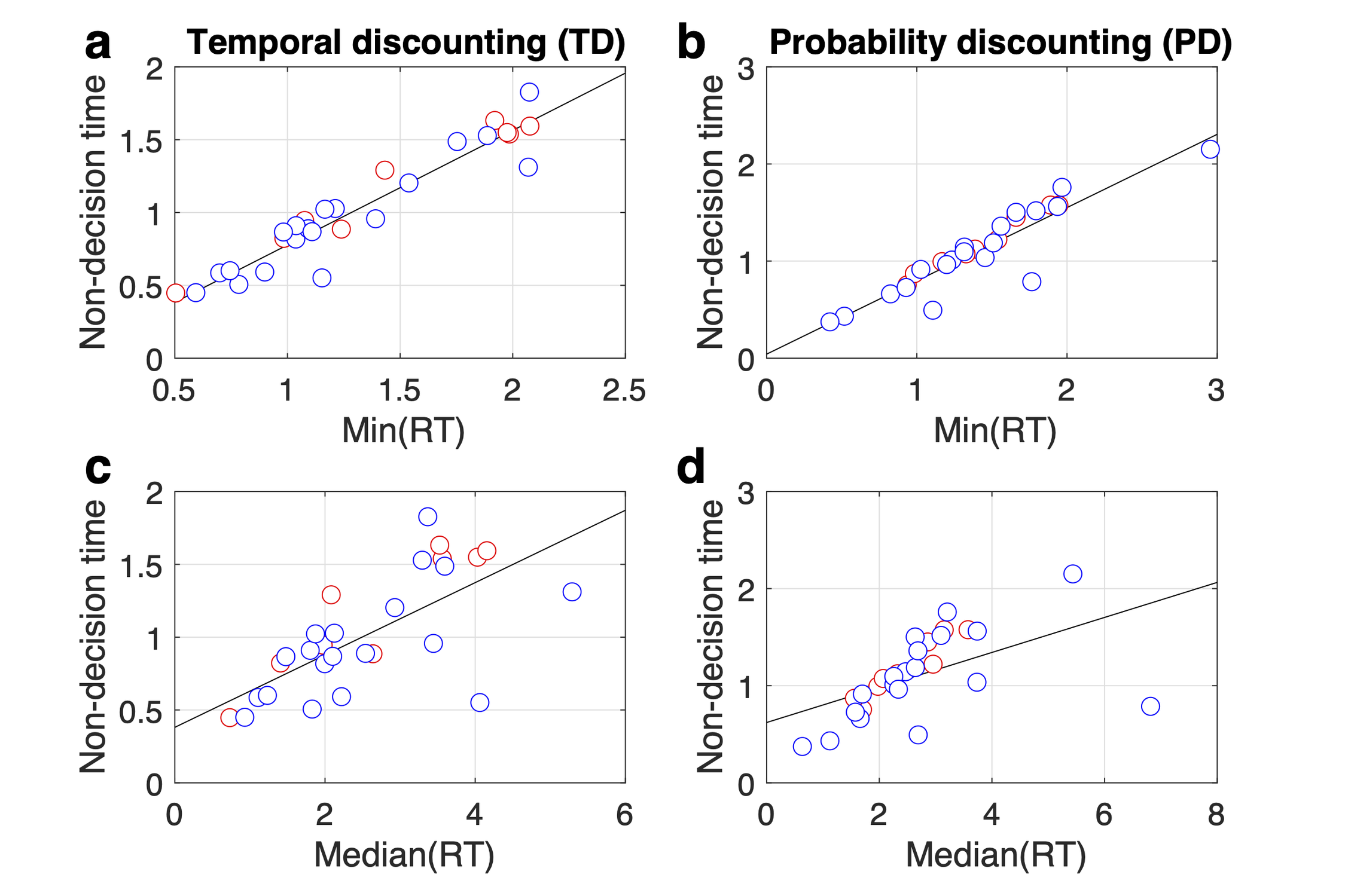

Supplement: S4 Fig — Scatter plots (red mOFC patients, blue: controls) depict associations between model-based non-decision time from the best fitting DDMS models (x-axis) and minimum RT (a/b) and median RT (c/d) for temporal discounting (a/c) and risky choice / proability discounting (b/d). (TIF) [file pcbi.1007615.s004.tif]

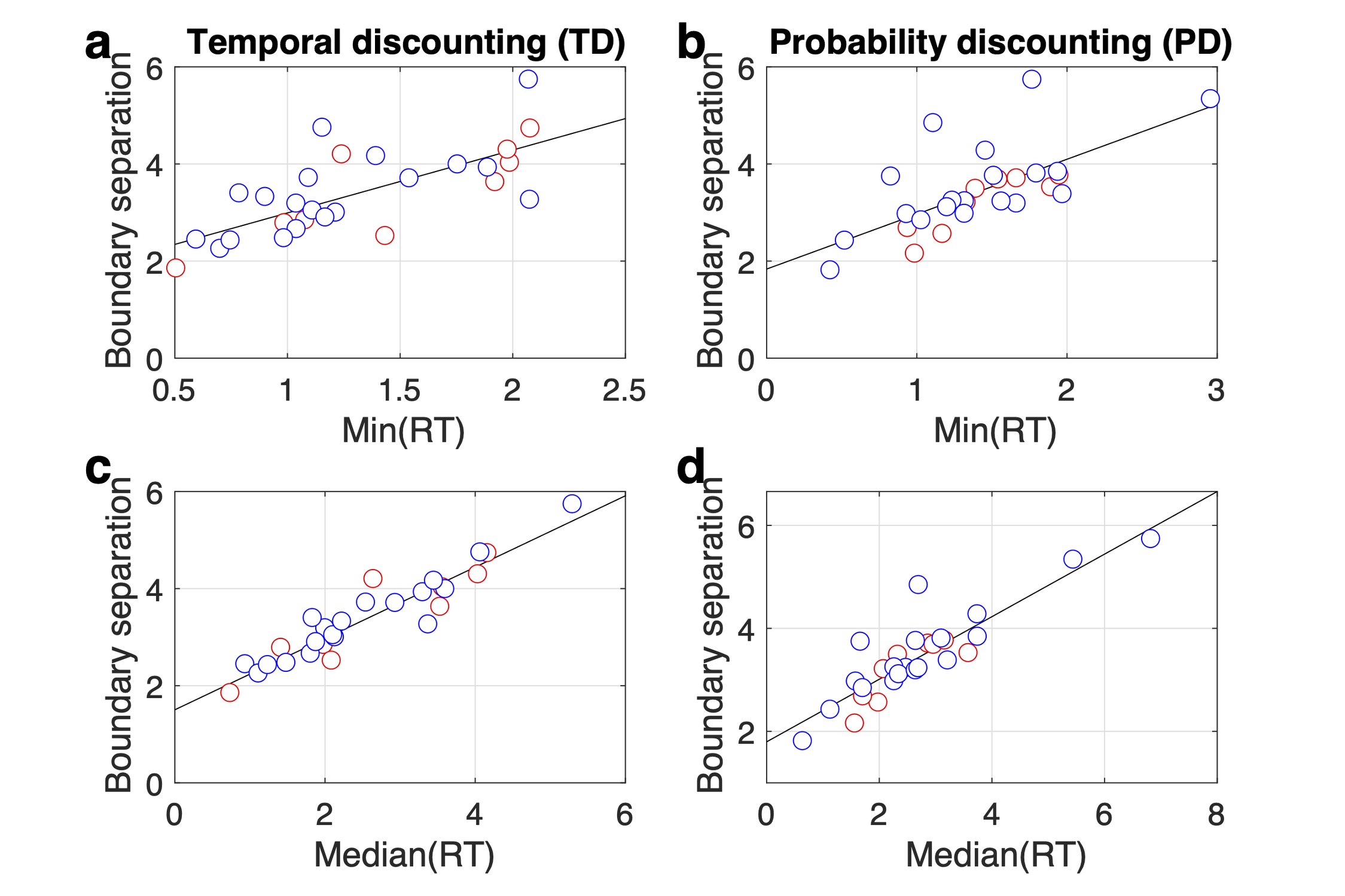

Supplement: S5 Fig — Scatter plots (red: mOFC patients, blue: controls) depict associations between model-based boundary separation from the best fitting DDMS models (x-axis) and minimum RT (a/b) and median RT (c/d) for temporal discounting (a/c) and risky choice / proability discounting (b/d). (TIF) [file pcbi.1007615.s005.tif]

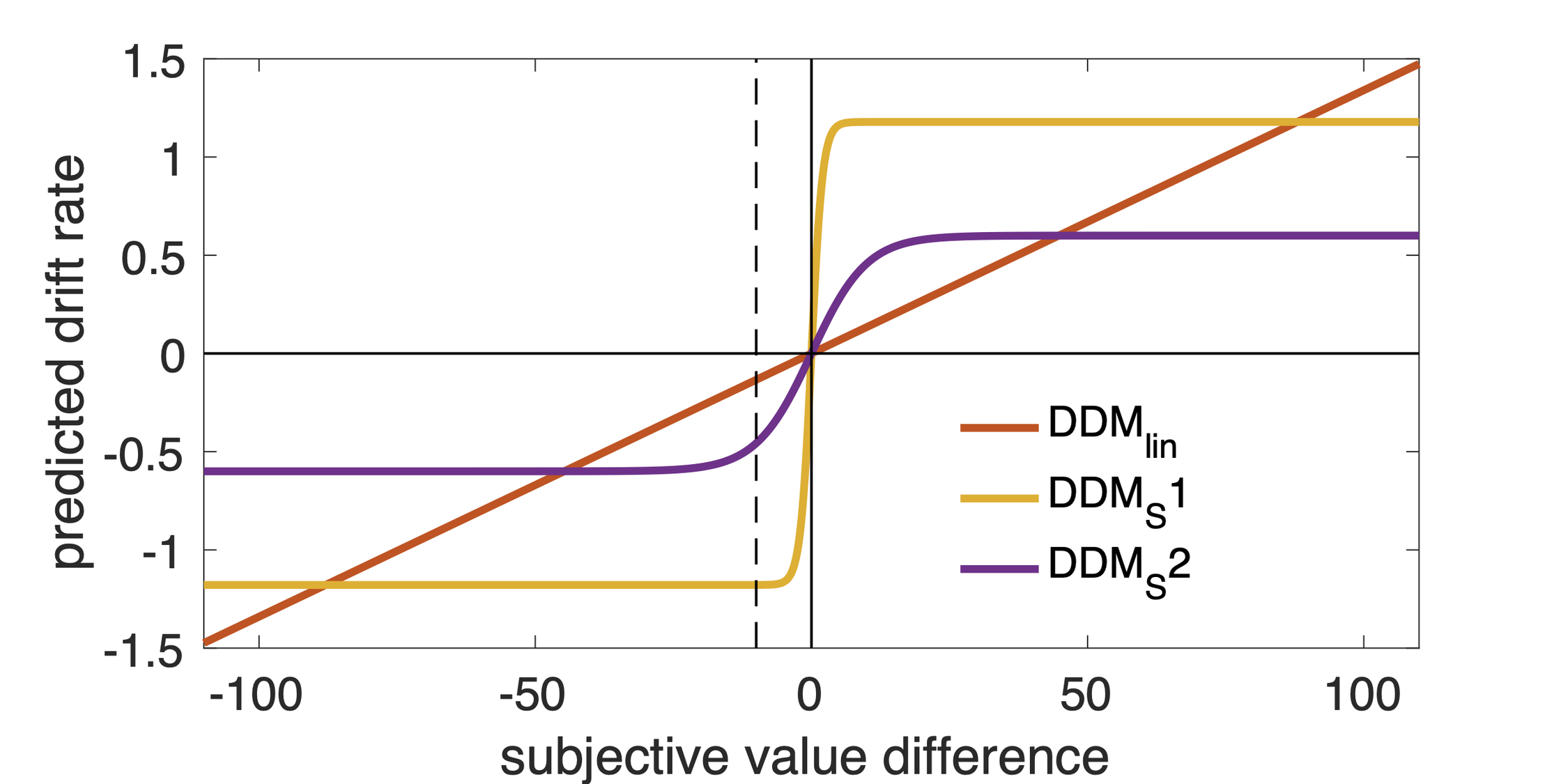

Supplement: S6 Fig — Linear scaling predicts longer RTs (lower drift rates) than sigmoid scaling for all but the greatest value differences, where the effect reverses. The reversal point depends on the drift rate components (DDMS1: vmax = 1.1786, vcoeff = .997, DDMS2: vmax = .6, vcoeff = .2). The dashed line marks a value difference of -10, which was the lower bound of value differences in the present experimental design (i.e., the case when the risky or larger-later option was discounted to almost 0). (TIF) [file pcbi.1007615.s006.tif]

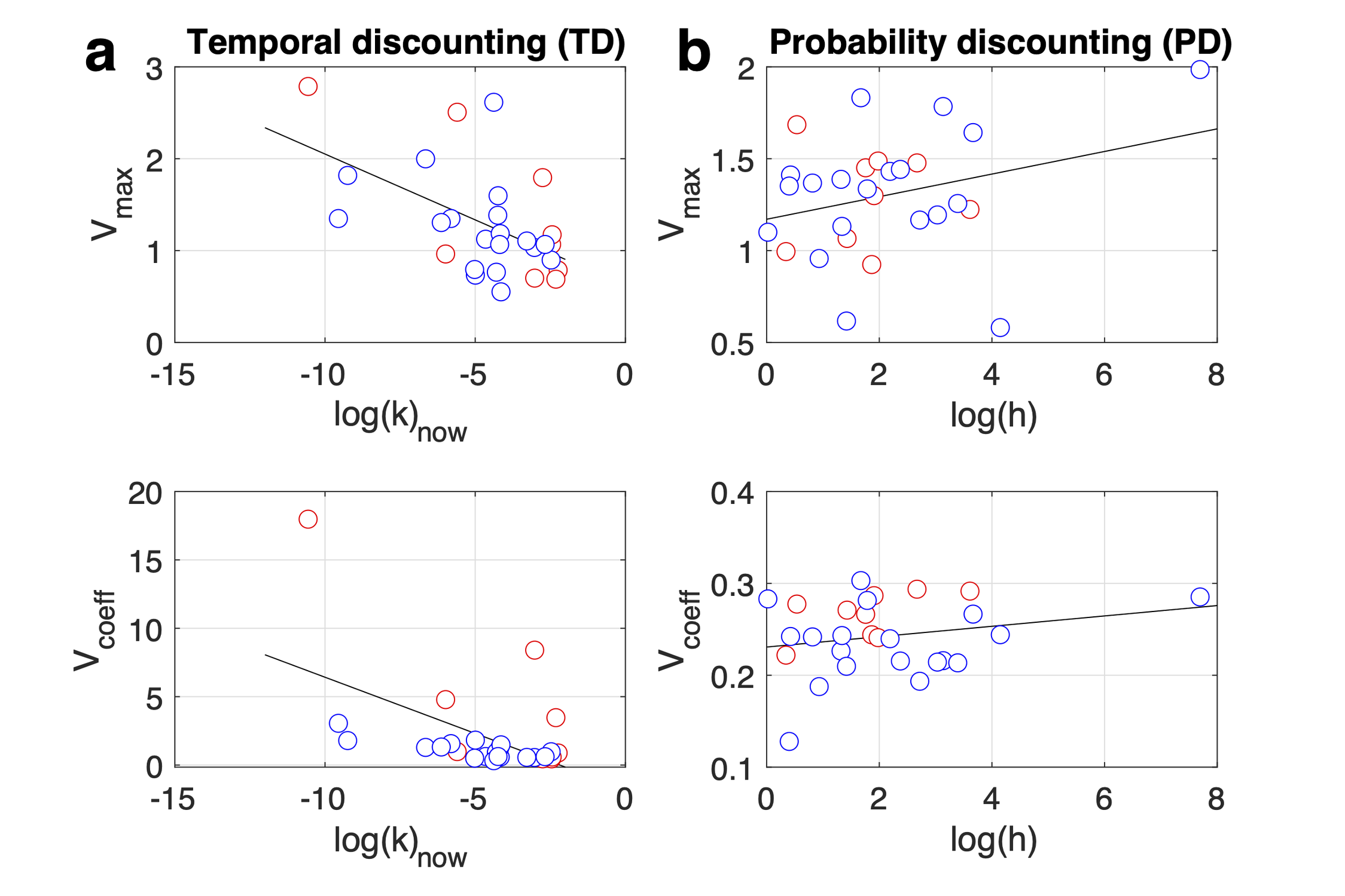

Supplement: S7 Fig — Associations between drift rate components and discount rates for temporal discounting (a) and risky choice / probability discounting (b). Top panels show vmax and lower panels show vcoeff. (TIF) [file pcbi.1007615.s007.tif]

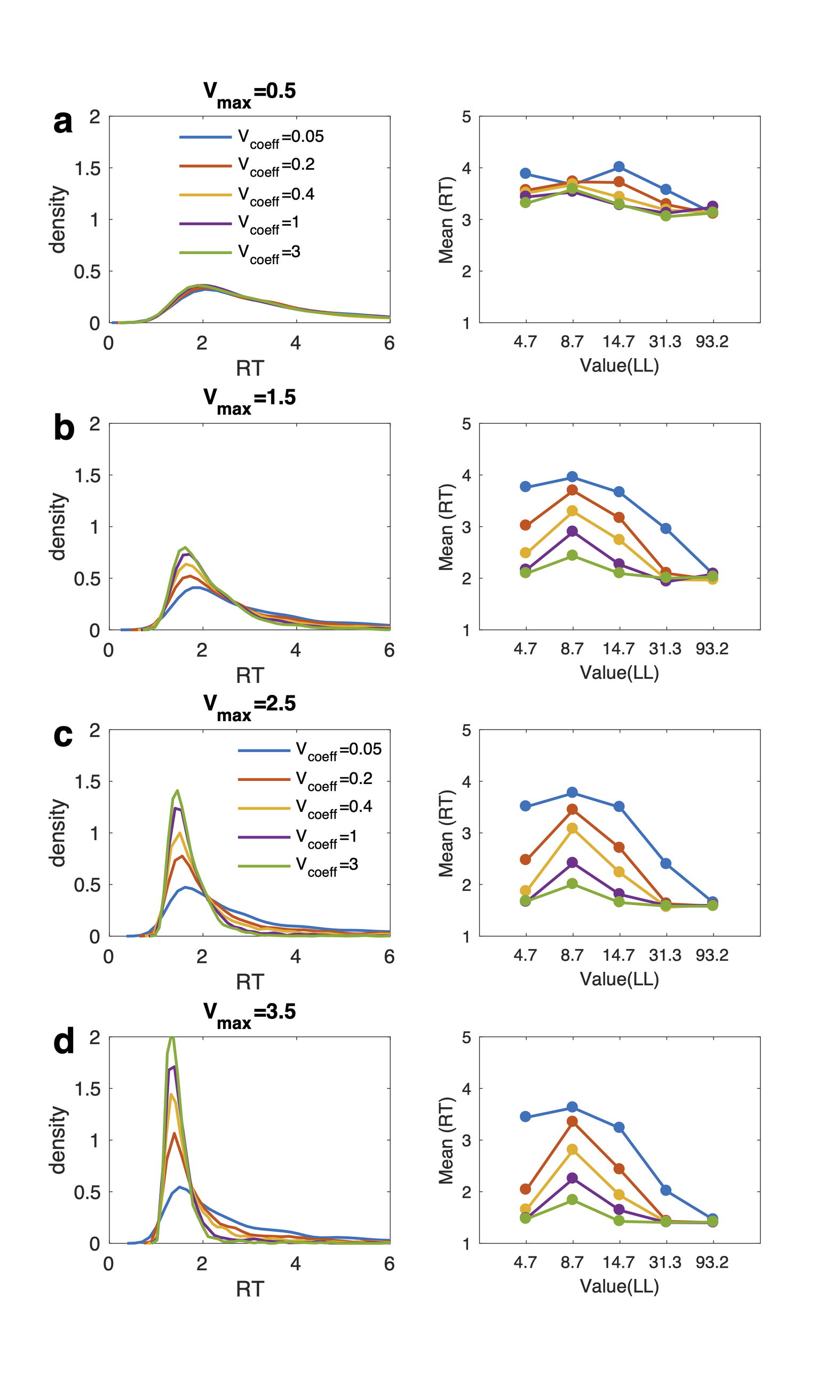

Supplement: S8 Fig — Simulated temporal discounting response time distributions (left) and mean predicted response times per value bin (right) for a virtual participant for different values of vmax and vcoeff. See S1 Table (left column) for parameter values. (TIF) [file pcbi.1007615.s008.tif]

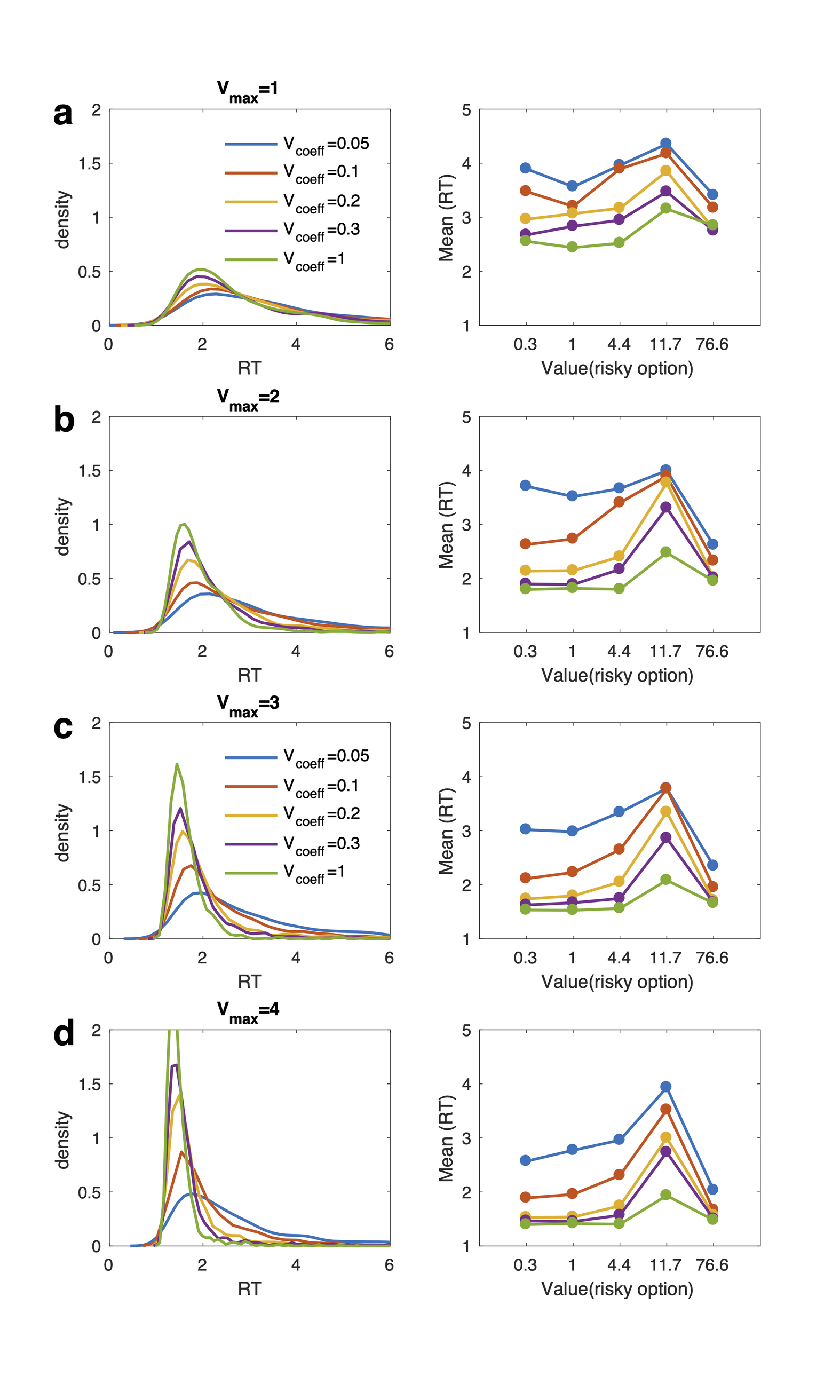

Supplement: S9 Fig — Simulated risky choice response time distributions (left) and mean predicted response times per value bin (right) for a virtual participant for different values of vmax and vcoeff. See S1 Table (right column) for parameter values. (TIF) [file pcbi.1007615.s009.tif]
