# Supplementary material for: The drift diffusion model as the choice rule in inter-temporal and risky choice: A case study in medial orbitofrontal cortex lesion patients and controls
Source: PLoS Comput Biol. 2020 Apr 20;16(4):e1007615. doi: 10.1371/journal.pcbi.1007615 (PMC7192518; doi:10.1371/journal.pcbi.1007615)
Supplement: S1 Table — All parameters are the posterior group means of the control group, with the exception of log(k)now and the two drift rate modulator variables, which were selected for illustrative purposes. (DOCX) [file pcbi.1007615.s010.docx]

|  | Temporal discounting | Risky choice |
| --- | --- | --- |
| Boundary separation (α) | 3.37 | 3.45 |
| Non decision time (τ) | .945 | .996 |
| Starting point / bias (*z*) | .531 | .461 |
| Drift rate *v* (max) | [.5, 1.5, 2.5, 3.5] | [1, 2, 3, 4] |
| Drift rate *v* (coeff) | [.05, .2, .4, 1] | [.05, .1, .2, .3] |
| Log(k)_now_ | -3.0 | - |
| Shift_log(k)_ | -.182 | - |
| Log(h) | - | 2.26 |
