# Supplementary material for: The drift diffusion model as the choice rule in inter-temporal and risky choice: A case study in medial orbitofrontal cortex lesion patients and controls
Source: PLoS Comput Biol. 2020 Apr 20;16(4):e1007615. doi: 10.1371/journal.pcbi.1007615 (PMC7192518; doi:10.1371/journal.pcbi.1007615)
Supplement: S1 Text — (DOCX) [file pcbi.1007615.s011.docx]

*Model checks: DDM_S_ parameters, softmax parameters and RT measures*

With the drift diffusion model as the choice rule, we introduced additional complexity, as softmax action selection typically only has a single free parameter ($\beta$). Therefore, we next examined the correspondence of choice model parameters (i.e. *log(k)_now_*, *shift_log(k)_* and *log(h)*) between models estimated using softmax vs. DDM_S_ choice rules. One would not expect preferences as reflected in these parameters to differ systematically as a function of whether only binary choices are fitted (softmax) or whether both choices and reaction times are jointly fitted (DDM_S_). A convergence between the estimated parameters from the two choice rules would therefore strengthen confidence in the applicability of the DDM in the context of the present tasks.

To this end, we extracted the mean single-subject parameter estimates for *log(k)_now_* (the hyperbolic discount rate in the *now* condition of the temporal discounting task, Eq. 1), *shift_log(k)_* (the parameter modeling the reduction in discounting between *now* and *not now* conditions in the temporal discounting task, Eq. 1) and *log(h)* (reflecting the degree of discounting of value over probabilities, Eq. 2) from the hierarchical fits of the two winning DDM_S_ models as well as from the hierarchical fits using standard softmax action selection. Figure S3 shows scatter plots of mean single-subject parameters estimated via softmax vs. via DDM_S_. Correlations were very high between the different choice rules (temporal discounting: *log(k)_now_* *r*=.93, *shift_log(k)_* *r*=.91; risky choice/probability discounting: *log(h)* *r*=.98). Since the correlation for *shift_log(k)_* appeared to be affected by the extreme data points in the mOFC patients, we re-ran the correlation separately for both groups, and observed significant positive associations in both cases (*r_controls_*=.52, *r_patients_*=.87). Together, these analyses confirm that parameters estimated via softmax modeling of binary choices can be reliably reproduced when jointly fitting choices and RTs via the DDM_S_.

Along similar lines, we next checked estimated DDM parameter against model-free RT statistics (minimum and median RT). The non-decision time $\tau$ captures RT components unrelated to the evidence accumulation process, and therefore reflects individual differences related to e.g. perceptual processing of the decision options and/or response preparation and execution. That is, for $\tau$ one would predict positive correlations in particular with the minimum RT, and to a lesser extent with median RT. As expected, correlations of $\tau$ with minimum and median RT where significant and more pronounced for minimum RT (Figure S4a,c: temporal discounting *r*_minRT_=.95, *r*_medianRT_=.69; Figure S4b,d: probability discounting *r*_minRT_=.92, *r*_medianRT_=.54). The boundary separation parameter $\alpha$ on the other hand reflects the threshold that the accumulated evidence needs to exceed before participants commit to a decision. Again one would expect positive correlations with minimum and median RT, but a more pronounced association with median RT. This is exactly what we observed (Figure S5a,c: temporal discounting *r*_minRT_=.71, *r*_medianRT_=.94; Figure S5b,d: probability discounting *r*_minRT_=.67, *r*_medianRT_=.88).
