# Supplementary material for: The drift diffusion model as the choice rule in inter-temporal and risky choice: A case study in medial orbitofrontal cortex lesion patients and controls
Source: PLoS Comput Biol. 2020 Apr 20;16(4):e1007615. doi: 10.1371/journal.pcbi.1007615 (PMC7192518; doi:10.1371/journal.pcbi.1007615)
Supplement: S2 Text — (DOCX) [file pcbi.1007615.s012.docx]

*Drift rate components v_max_ and v_coeff_ and discounting behavior*

We also explored the association between drift rate components and discounting behavior. S7 Figure plots the discount rates log(k) and log(h) against these parameters (means of individual subject posterior distributions for each parameter). Uncorrected non-parametric Spearman-correlation coefficients where as follows: ρ_log(k), vmax_ =-.507, *p*=.0065; ρ_log(k), vcoeff_ =-.432, *p*=.0227; ρ_log(h), vmax_ =.216, *p*=.269; ρ_log(h), vcoeff_ =.138, *p*=.482.
