# Supplementary material for: The drift diffusion model as the choice rule in inter-temporal and risky choice: A case study in medial orbitofrontal cortex lesion patients and controls
Source: PLoS Comput Biol. 2020 Apr 20;16(4):e1007615. doi: 10.1371/journal.pcbi.1007615 (PMC7192518; doi:10.1371/journal.pcbi.1007615)
Supplement: Attachment — Submitted filename: R1_ResponseLetter_Peters.pdf [file pcbi.1007615.s013.pdf]

Peters & D'Esposito (2019): The drift diffusion model as the choice rule in inter-temporal and risky decision-making: a case study in medial orbitofrontal cortex lesion patients and controls.

Response to Reviewer's Comments

We'd like to thank the two reviewers for their thoughtful and detailed comments. We have carried a range of additional analyses and simulations as well as comprehensive parameter recovery analyses. We believe that the paper is substantially improved and hope that the changes meet your approval.

Reviewer #1: In the manuscript entitled "The drift diffusion model as the choice rule in inter-temporal and risky choice: a case study in medial orbitofrontal cortex lesion patients and controls", Peters & D'Esposito present some novel insights by fitting cognitive models to partly unpublished data. These insights include: (1) vmPFC/mOFC damage patients have longer non-decision times and reduced decision caution compared to healthy and age- and education-matched controls; (2) they show increased risk-taking; (3) the best way to account for value-based decisions in an inter-temporal choice task as well as in a risk-taking task is to modify the DDM so that the rate of evidence accumulation is proportional to the difference in subjective value between the two options; (4) the mapping between value-differences and the rate of evidence accumulation is non-linear rather than linear.

The present manuscript offers new insights in addition to the current literature in value-based decision making. However, there are a number of points (major and minor ones) that I think should be addressed by the authors.

Major points:

To test the hypothesis that the "vmPFC/mOFC damage might also render RTs during decision-making less dependent on value" it would be better to use a mixed-modelling approach rather than simply looking for differences in the drift-value-coefficient between groups of participants. This is because a low vs. high drift-value-coefficient means that decisions are still based on values, but are less sensitive to value differences. What would be interesting to see, is whether for a higher portion of trials compared to control subjects participants with vmPFC/mOFC damage can be simply described by the null-DDM instead of by the value-DDM. This could be tested with a mixed model, where a parameter (e.g., lambda) could control the proportion of trials that can be best described by a null-DDM vs. a value-DDM. This would allow to formulate and test the hypothesis that the lambda would be higher in participants with a vmPFC/mOFC damage.

Response: Thank you, this is a great idea. We carried out the suggested mixture model analysis in the following way: For each task, we fit an additional model that included full parameter sets of both the DDM_0 and the DDM_S. We then included an additional mixture parameter lambda for each participant that modeled the proportion of trials accounted for by the DDM_S. Group posterior distributions for lambda are shown below. In z-units, lambda was 2.2 and 2.58 in mOFC patients and controls, respectively, for the temporal discounting data, and 2.77 and 2.57 for the risky choice data. Thus, on average, in both groups >98% of trials were better accounted for by the DDM_S compared to the DDM_0. Furthermore, difference distributions were centered at zero in both cases, and Bayes Factors revealed little evidence

for a group difference (.5 < BF < 2.1). The new results section on the mixture models (p. 15) reads as follows:

DDM mixture models

Both the model comparison and the posterior predictive checks suggest that choices in vmPFC/mOFC patients were still modulated by value. But the simulations showed that both very high and very low values of v_{coeff} can produce RTs that are more uniform across value differences – RTs tend to be more uniformly fast for high values of v_{coeff}, and more uniformly slow for low values. Therefore, we additionally ran a more direct test of value sensitivity following vmPFC/mOFC damage by setting up DDM mixture models (see methods section). In short, these models allowed a proportion of trials to be produced by the DDM_0 and the remaining trials to be produced by the DDM_S, with an additional free parameter λ controlling the mixing proportion. Notably, this analysis is agnostic with respect to the directionality of potential changes in v_{max} and v_{coeff}, and instead solely focuses on whether groups differ in the proportion of trials produced by a value-DDM vs. the DDM_0. Posterior distributions for λ are shown in Figure 9. For this analysis, λ was estimated in standard normal space and transformed to the interval [0, 1] via an inverse probit transformation on the subject level. In *z*-units, the posterior group mean of lambda was 2.2 and 2.58 in mOFC patients and controls for the temporal discounting data (Figure 9a), and 2.77 and 2.57 for the risky choice data (Figure 9b). Thus, on average, in both groups >98% of trials were better accounted for by the DDM_S compared to the DDM_0. Difference distributions were centered at zero in both cases, and Bayes Factors revealed little evidence for a group difference (.5 < BF < 2.1).

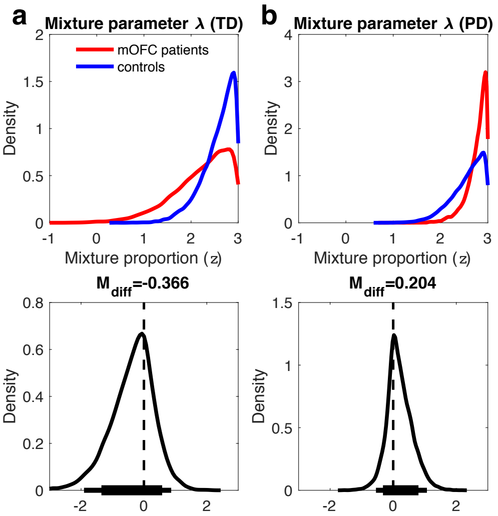

Figure 9. Top row: posterior distributions of the mixture parameter λ (a: temporal discounting (TD), b: risky choice / probability discounting (PD)) in z-units. Positive values of λ indicate that a greater proportion of trials was better accounted for by DDM_S vs. DDM_0, whereas negative values indicate the reverse. λ was fitted in standard normal space with a group-level uniform prior of [-3, 3] and backtransformed on the subject-level via an inverse probit transformation. Bottom row: Posterior group differences (mOFC patients – controls) for each parameter. Solid horizontal lines indicate highest density intervals (HDI, thick lines: 85% HDI, thin lines: 95% HDI).

Reviewer #1: It seems to me that the null model (DDM0) is too simple, making the comparison with the two value-based DDMs unfair. I strongly suggest to fit separate sets of the 4 parameters (alpha, tau, v, and z) across conditions (Now vs. Not now) for the inter

temporal choice task. Since there are no clear conditions in the risky choice task, I am not sure how this could be done there.

Response: We agree that it might be informative to further explore condition-related differences in the DDM parameters for the inter-temporal choice task. However, such a DDM0 model would need to be compared to value-based DDMs that similarly allow all DDM parameters (alpha, beta, tau, vcoeff, vmax) to differ between conditions. Given the extensive revisions and simulations that we have already included in this revised version (see additional analyses below) and that have considerably expanded the paper, we have decided to refrain from examining condition effects in the temporal discounting task further.
We have added a section to p. 18 of the discussion to address this issue, where we now write:

The temporal discounting task, but not the risky choice task, was comprised of two experimental conditions (immediate vs. delayed smaller-sooner rewards). However, we have refrained from examining condition differences in the DDM parameters in greater detail, and instead only modeled a shift parameter for log(k), rather than for the full set of DDM parameters. This was done for simplicity and in order to keep analyses comparable between tasks. However, how contextual factors and framing effects[60,61] impact choice dynamics during inter-temporal and risky choice will be an interesting future avenue for research.

Reviewer #1: Regarding the prior distributions, I have 2 suggestions: (1) that the priors are properly written and described in the text or appendix, or in the supplementary materials. They should be easily accessible by a reader and should not just be retrievable within the online code. Note that it is also necessary to specify the priors for the individual parameters, depending on the group parameters; (2) uniform priors are not uninformative, especially when restricted to sensible ranges. This suggests that the authors had an idea of which values were sensible and could therefore specify weakly-informative priors (see Gelman et al., 2014). I suggest to use "sensibly" centered Cauchy distributions – since they have the advantage of being heavy-tailed distributions and therefore weakly-informative – for the means and Half-Cauchy for the standard deviations.

Response: Thanks for this suggestion. Regarding (1), we have re-written the modeling section in the methods section and report all requested information regarding the prior distributions in Table 7 and the methods section on Hierarchical Bayesian Models. Regarding (2), we appreciate that opinions regarding group-level priors vary amongst researchers. Given that we are investigating not only elderly controls but also lesion patients, it is not clear where Cauchy distributions for the different group level means should "sensibly" be centered. Therefore, in our view uniform priors across large (but numerically plausible) regions of the parameter space are a straightforward way to not bias results. We agree that the term "sensible ranges" that we used previously is misleading, and we now instead write "numerically plausible ranges", which better describes our approach. We agree however that such priors are not uninformative, and this term was therefore removed.

Reviewer #1: The authors should add a parameter recovery section for the winning model (as in, e.g., Pedersen et al., 2017, Fontanesi et al., 2019a, and Fontanesi er al., 2019b). This is quite crucial when proposing new, complex models such as value based modifications of the DDM.

Response: This is a very good point. In the revised version of the paper, we now include an extensive section on parameter recovery, in accordance with this suggestion.
On p. 10, we now write:

Parameter recovery simulations

A further crucial property of a model is that parameters are identifiable, such that if generating parameters are known, they should be recoverable. As done in previous work[14,15] we therefore carried out parameter recovery analyses for the most complex model (DDM_S). Ten simulated data sets were randomly selected (see methods section) and re-fit using the DDM_S. We then compared the generating (true) parameter values to the estimated values. Subject-level parameters generally recovered well (Figure 6a and Figure 7a). Group level means and standard deviations (calculated based on the precision) generally also recovered well (Figure 6b-e, Figure 7b-e), such that in most cases, the 95% highest density intervals of the estimated posterior distributions included the true generating parameter values. For parameters that showed a high variance (e.g. v_{coeff} and $log(k)_{now}$ in the patient group) the group-level standard deviations tended to be overestimated.

In short, we re-fit the DDM_S model to 10 datasets simulated from the posterior distribution, and examined parameter recovery for both individual-level (top row scatter plots in the Figures below) and group-level parameters (bottom rows of the plots, with squares denoting true group-level parameters and lines denoting 95% HDIs of the posteriors):

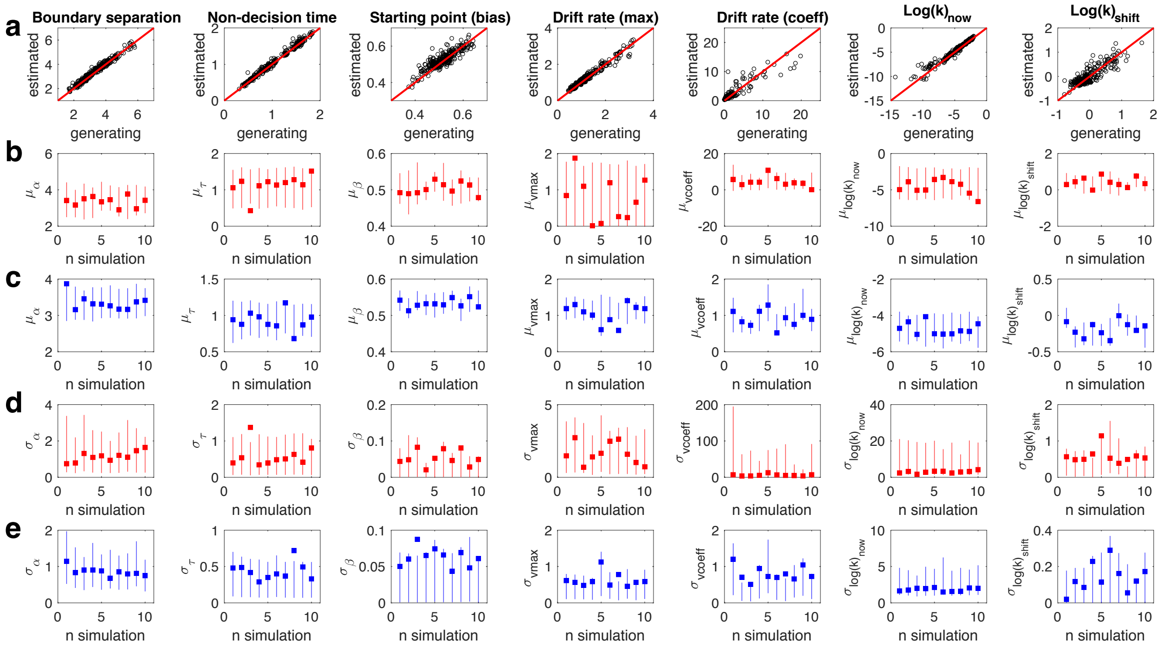

Figure 5. Parameter recovery results for the temporal discounting DDM_S. a: Recovery of subject-level model parameters pooled across all ten simulations. b/c: true generating group level means (squares) for mOFC patients (b, red) and controls (c, blue) and estimated 95% highest density intervals (lines) per simulation. d/e: generating group level standard deviations (squares) for mOFC patients (d, red) and controls (e, blue) and estimated 95% highest density intervals (lines) per simulation.

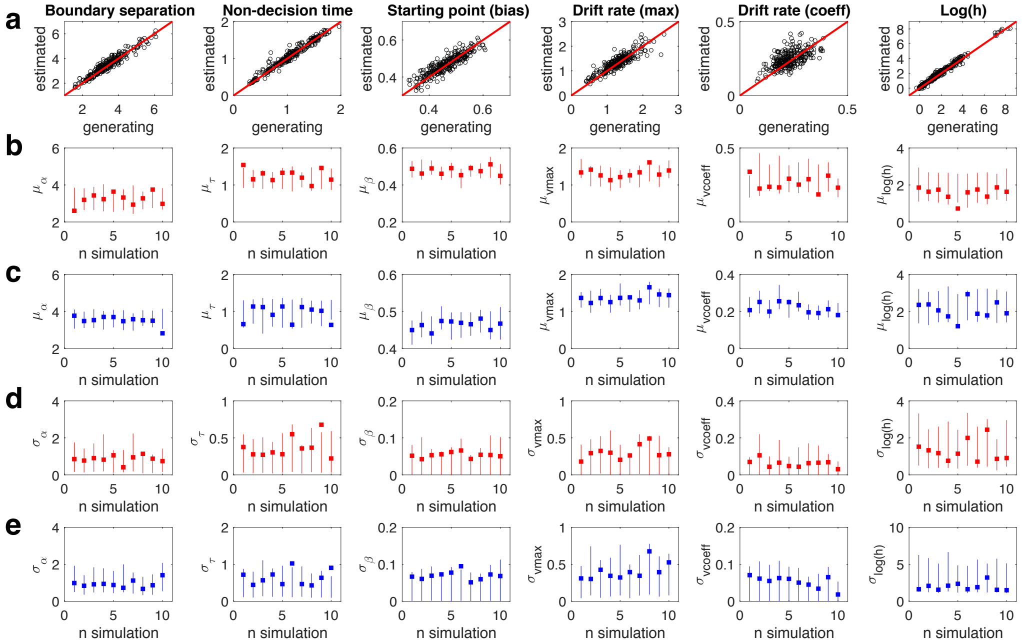

Figure 6. Parameter recovery results for the risky choice DDM_S. a: Recovery of subject-level model parameters pooled across all ten simulations. b/c: generating group level means (squares) for mOFC patients (b, red) and controls (c, blue) and estimated 95% highest density intervals (lines) per simulation. d/e: generating group level standard deviations (squares) for mOFC patients (d, red) and controls (e, blue) and estimated 95% highest density intervals (lines) per simulation.

Reviewer #1: The posterior predictive checks are hard to assess. To better assess them, the authors should group mean RTs or RT quantiles for SS vs. LL options or for risky vs. safe options by condition and compare them with the same summary statistics on the data (as in, e.g., Fontanesi et al., 2019a, Fontanesi et al., 2019b)

Response: Thanks, this is a very important point that resonates with a comment made by Reviewer #2, who asked for additional evidence that the DDM_S not only better accounted for binary choices but also for RT distributions. Given that the superior fit of the DDM_S suggests that how value impacts RTs plays a central role in which model accounted for the data best, we used a modification of the approach suggested by Reviewer #1. That is, we grouped trials per subject into five value bins based on the subjective discounted value of the LL (or risky) reward. Note that in this analysis, one would expect the longest RTs for bins with a mean subjective value closer to 10 (the constant reference reward value in both tasks) for the value-based models. In contrast, the null model predicts the same RTs regardless of values. We then plotted the mean RTs for each value bin against the RTs predicted by each DDM model (see below). Generally, the DDM_S accounted for the value-modulation of RTs much better than the DDM_{lin} in the majority of participants:

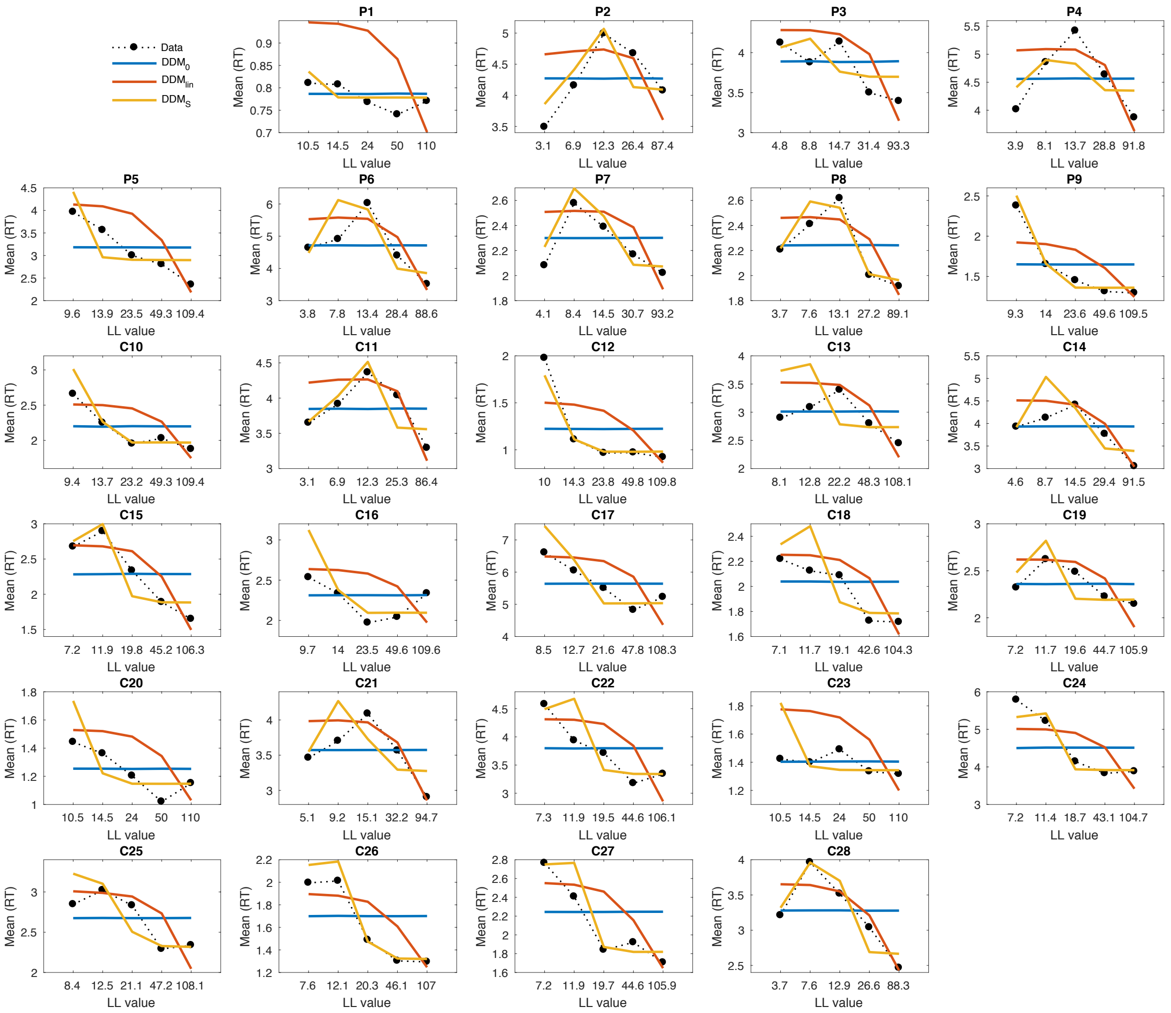

Figure 2. Posterior predictive plots for the different temporal discounting DDM models for all individual participants (P – mOFC patients, C – controls). Trials were binned into five bins of equal sizes according to the subjective value of the larger-later (LL) option for each participant (calculated according to equation 1). The x-axis in each panel shows the subject-specific mean LL value for each bin. The y-axis denotes observed response times per bin (dotted black lines) and model predicted response times per bin for the different DDM models (blue: DDM_0, red: DDM_{lin}, orange: DDM_S). Model predicted response times were obtained by averaging over 10k data sets simulated from the posterior distribution of each hierarchical model.

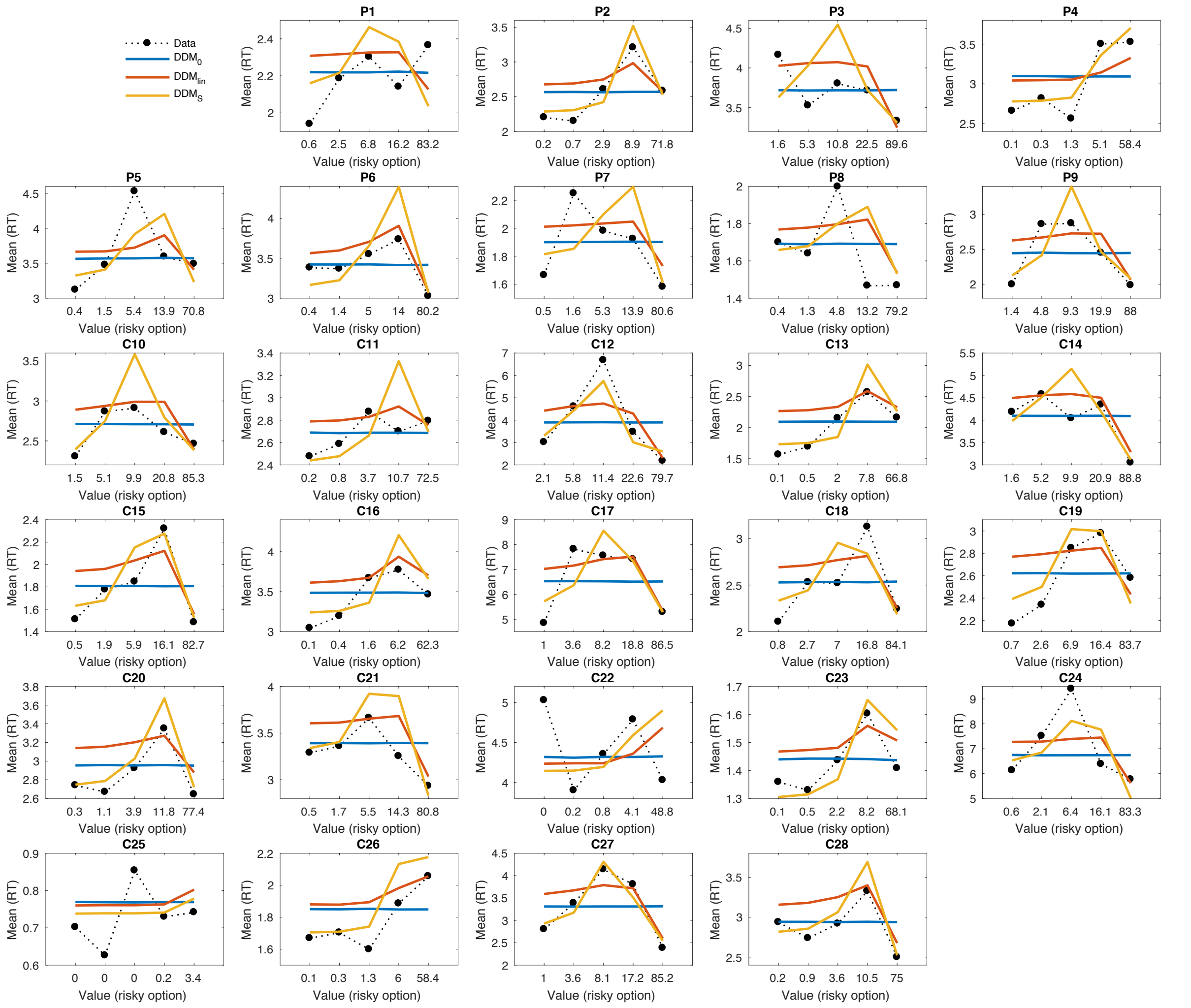

Figure 3. Posterior predictive plots for the different risky choice DDM models for all individual participants (P – mOFC patients, C – controls). Trials were binned into five bins of equal sizes according to the subjective value of the risky option for each participant (calculated according to equation 2). The x-axis in each panel shows the subject-specific mean LL value for each bin. The y-axis denotes observed response times per bin (dotted black lines) and model predicted response times per bin for the different DDM models (blue: DDM_0, red: DDM_{lin}, orange: DDM_S). Model predicted response times were obtained by averaging over 10k data sets simulated from the posterior distribution of each hierarchical model.

The corresponding paragraph in the Results section (p. 7) now states:

In a second step, we directly compared the ability of the DDM_S and DDM_{lin} to account for how value modulates RTs. To this end, we binned trials for each subject into five bins according to the subjective value of the LL or risky reward according to equations 1 and 2. We then simulated 10k full data sets from the posterior distributions of each model (DDM_0, DDM_{lin}, DDM_S) and averaged model predicted response times per bin. Results are shown for each participant in Figure 2 for temporal discounting and Figure 3 for risky choice. The DDM_0 does not incorporate values, thus it predicts the same RTs across value bins (horizontal blue lines in Figure 2 and 3). While the DDM_{lin} could account for some aspects of the association between values and response times in some participants, the DDM_S provided a much better account of this relationship overall.

Reviewer #1: For the Cohen's d calculation, the authors should use the whole posterior traces for mean and pooled standard deviation, instead of the mean of the posterior distributions. This would make the calculation "more Bayesian" and more reliable.

Response: I am not sure I understand. We used the means of the whole posterior traces to calculate means and pooled standard deviation to derive an effect size point estimate for future reference and comparison to other studies. But this measure is reported for illustrative and future reference purposes only, not for inference.

Reviewer #1: I would exclude from the analyses correlations between DDM parameter estimates and model-free RT statistics. The DDM is supposed to decompose RT and choices distributions into interpretable parameters so: (1) some parameters always correlate to some extent to RTs (2) it is unclear to me what this would add to the interpretation of the model. If the aim was to check for qualitative model fit, the posterior predictive checks should be enough.

Response: Thanks, as a similar point was raised by Reviewer #2 (see below), we re-labled these analyses "sanity checks" and moved them into the SI. The main text of the manuscript now only contains the following statement on p. 5:

Model validation
We first carried out a number of simple sanity checks (see S1 Text) which confirmed that log(k) parameters estimated via standard softmax and via the DDM_S showed good correspondence (S3 Figure). Likewise, minimum and median RT showed the expected associations with model-based non-decision times (S4 Figure) and boundary separation parameters (S5 Figure).

Minor points:
Reviewer #1: Please substitute every recurrence of "reaction time" with "response time", which is more appropriate in this case (as this is not a mere stimulus reaction task but participants need to integrate information in order to make a decision). In general, I would only write response time (RT) at the first recurrence, and only refer to it as RT or RTs after that.

Response: This has been corrected.

Reviewer #1: Regarding model comparison, I would suggest to switch to WAIC or LOO, which also allow you to have an estimate of the error in such measures and more reliably assess the difference in model fit between two models (as in, e.g., Pedersen et al., 2017 and Fontanesi et al., 2019a). This can be easily achieved via the R loo package, that only needs the mcmc traces as input (you could save your analyses output from Matlab and load them in R…).

Response: Thanks, good point. We followed this suggestion and reproduced the exact same model ranking that we observed for DIC using these alternative measures. These are now reported in Tables 1 and 2, where we report WAIC and the estimated log pointwise predictive density (elpd), as well as a 95% CI for the differences in elpd:

Table 1. Model comparison of drift diffusion models of temporal discounting. The hyperbolic+shift value function (see Eq. 1) corresponds to hyperbolic discounting in the *now* condition, and a shift parameter that models the decrease in discounting between the *now* and *not now* conditions. WAIC – Widely Applicable Information Criterion; elpd – estimated log predictive density; $elpd_{diff}$ is the difference in elpd between each model and the DDM_S.

Model	Drift rate scaling	Value function	WAIC	-elpd	$-eldp_{diff}$ [95% CI]
DDM_0	-	-	20939	10472.5	1987.9 [1899.1 – 2076.7]
DDM_{lin}	*Linear*	*Hyperbolic+Shift*	19602	9805.2	1320.6 [1231.2 – 1409.9]
DDM_S	*Sigmoid*	*Hyperbolic+Shift*	16966	8484.6	-

Table 2. Model comparison of drift diffusion models of risky choice. The hyperbolic value function (see Eq. 2) corresponds to hyperbolic discounting over the odds-against-winning the gamble. WAIC – Widely Applicable Information Criterion; elpd – estimated log predictive density; $elpd_{diff}$ is the difference in elpd between each model and the DDM_S.

Model	Drift rate scaling	Value function	WAIC	-elpd	$-eldp_{diff}$ [95% CI]
DDM_0	-	-	11515	5760.3	1162.8 [1094.4 – 1231.2]
DDM_{lin}	*Linear*	*Hyperbolic*	10422	5222.4	625.0 [546.0 – 703.9]
DDM_S	*Sigmoid*	*Hyperbolic*	9190	4597.4	-

Reviewer #1: I do not understand what it means that the distributions should have a clear peak or a clearly Gaussian shape: not all parameter distributions are supposed to have such a shape, so this shouldn't be a way to asses model recoverability. On the contrary, looking at chain convergence and parameter recovery are.

Response: This statement has been corrected.

Reviewer #1: The "m" in Equation 7 is not the slope of the sigmoid function, as described in the main text, but is the input value-difference that is transformed by the sigmoid function. The slope would then be Vcoeff (Vmod in Fontanesi et al., 2019a). R-hats statistics should just be between 1 and some value close to 1. So please correct to 1 <= Rhat <= 1.01

Response: These points have been corrected.

Reviewer #1: In the results, clarify this sentence: "Since the correlation for shiftlog(k) appeared to be somewhat inflated by the extreme datapoints of the mOFC patients, we re-ran the correlation only in the control group. Here, the correlation was lower but still robust (r=.52)." What are these extreme datapoints? What was the correlation in the mOFC patients?

Response: We have moved these "sanity check" analyses into the supplement, and clarified the sentence. The "extreme data points" are the high parameter values in the patient group. In the S1 Text, we now write:

Since the correlation for $shift_{log(k)}$ appeared to be affected by the extreme data points in the mOFC patients, we re-ran the correlation separately for both groups, and observed significant positive associations in both cases ($r_{controls}$=.52, $r_{patients}$=.87).

Reviewer #2: Very minor points:In the abstract, refer to “Bayesian parameter estimation” instead of “Bayesian estimation scheme”.In the introduction, remove the word “usually” in the first sentence of the second paragraph. DDM can only have 2 response boundaries, otherwise it would be a different model. In the last sentence of the same paragraph substitute “simple” with 2-alternatives forced choice tasks (this are what the DDM was made for). I think it’s a bit misleading to call the DDM boundaries 0 and 1, so if possible I would delete that, or better explain that the lower boundary corresponds to when the accumulated evidence is equal to 0, the upper boundary is who the accumulated evidence is equal to alpha and the starting point z is half alpha.Put a comma after every recurrence of e.g. (e.g.,).

Response: These points have been corrected.

Reviewer #2: In the current study, the authors are using drift-diffusion modeling to predict a combination of choice and RT data, from two reinforcement learning tasks (temporal/probability discounting) performed by healthy controls and mOFC/vmPFC lesion patients. The authors suggest that DDM can be adequately used to describe observed data, and that it does not fall behind compared to a more conventional RL model, describing only choice behavior. The authors then suggest that (a) a ddm with a non-linear mapping between subjective-value and drift-rate provide the best fit to the data compared to more conventional ddm models. (b) Group differs mainly in increased non-decision time, and reduced decision threshold for patients vs. controls. The authors report no group differences in value processing.

I believe this is a valuable study, both in the sense that it aims to contribute by examining the applicability of evidence accumulation modeling to described choice&RT data, and presents interesting results with lesion patients. However, I think there are some issues that needs to be further elaborated and explored:

1. The authors conclude that non-linear drift rate modulation provided the best fit to the data. This is a great finding, but I think that it is very important to understand why that is, in the mechanistic level, specifically in terms of the relationship between subjective value and decision-time:

a. At the moment, it is hard to figure out why the non-linear aspect of the DDMs allows a better description for the data. Is this because the RT association with value differences between the two options is stronger for lower value differences? Or maybe this is due to the fact that some participants are less sensitive to the value manipulation (i.e., resulting in a very high Vcoef)?

b. Does the better fit for DDMs vs. DDMlin comes only from choice data, or does it provide better fit to RT data as well? I believe this is important to fully understand what part of the observed data is better explained by DDMs (e.g., it might not be about RTs at all, with DDMs better accounting for choice data alone compared to DDMlin). If it is mostly due to choice data, I am not sure why the use of ddm is justified here.

Response: These are very good points, thanks. We addressed these issues in the following way: First, in Tables 3 and 4 (see below) we now report the raw proportion of correctly predicted binary choices for each model (Softmax, DDM_{lin} and DDM_S). Arcsine-Sqrt-Transformed accuracy values used for statistical analysis are plotted in Figure 2 (see below). These analyses show that indeed DDM_S provides a significantly better account of binary temporal discounting choices than DDM_{lin}. For risky choice, the effect was in the same direction but not significant. The corresponding section on p. 6 now reads:

Prediction of binary choice data

We then checked the degree to which the different implementations of the DDM predicted participants' binary choices. We used each participant's mean posterior parameters from the hierarchical models to calculate model predicted choices, and compared these to the observed binary choices. The raw accuracy scores per model and group are listed in Table 4 (temporal discounting) and Table 5 (risky choice) with the softmax models shown for comparison. Numerically, accuracy scores for the DDM_S were higher than for DDM_{lin}. Indeed variance-stabilized accuracy values (arcsine-square-root transformed, see Figure 2) were greater for DDM_S compared to DDM_{lin} for temporal discounting (t_{27}=-7.43, 95% CI: [-.19, -.11]), with a similar trend for risky choice (t_{27}=-1.97, 95% CI: [-.09, .002]).

Table 3. Median (range) of the proportion of correctly predicted binary choices for the different temporal discounting models, separately for mOFC patients and controls.

	**Softmax**	**DDM$_{lin}$**	**DDM$_S$**
**mOFC patients**	.92 (.87-.99)	.90 (.80-.96)	.92 (.89-.99)
**Controls**	.91 (.78-.96)	.75 (.60-.99)	.91 (.84-.99)

Table 4. Median (range) of the proportion of correctly predicted binary choices for the different risky choice models, separately for mOFC patients and controls.

	**Softmax**	**DDM$_{lin}$**	**DDM$_S$**
**mOFC patients**	.92 (.82-.97)	.90 (.82-.95)	.92 (.84-.98)
**Controls**	.92 (.82-.99)	.91 (.79-.99)	.91 (.82-.99)

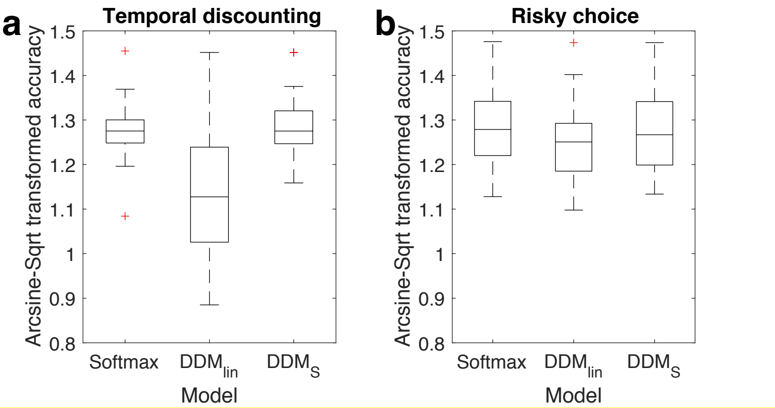

Figure 1. Variance-stabilized proportion of trials (arcsine-square root transformed) on which each model correctly predicted binary decisions for temporal discounting (a) and risky choice (b).

Second, we improved the posterior predictive checks to address the other issue raised by this Reviewer, i.e. whether also RTs were better accounted for by the DDM$_S$. Here we now plot for each subject the observed and model predicted RTs as a function of the subjective value of the LL or risky option e.g., for temporal discounting:

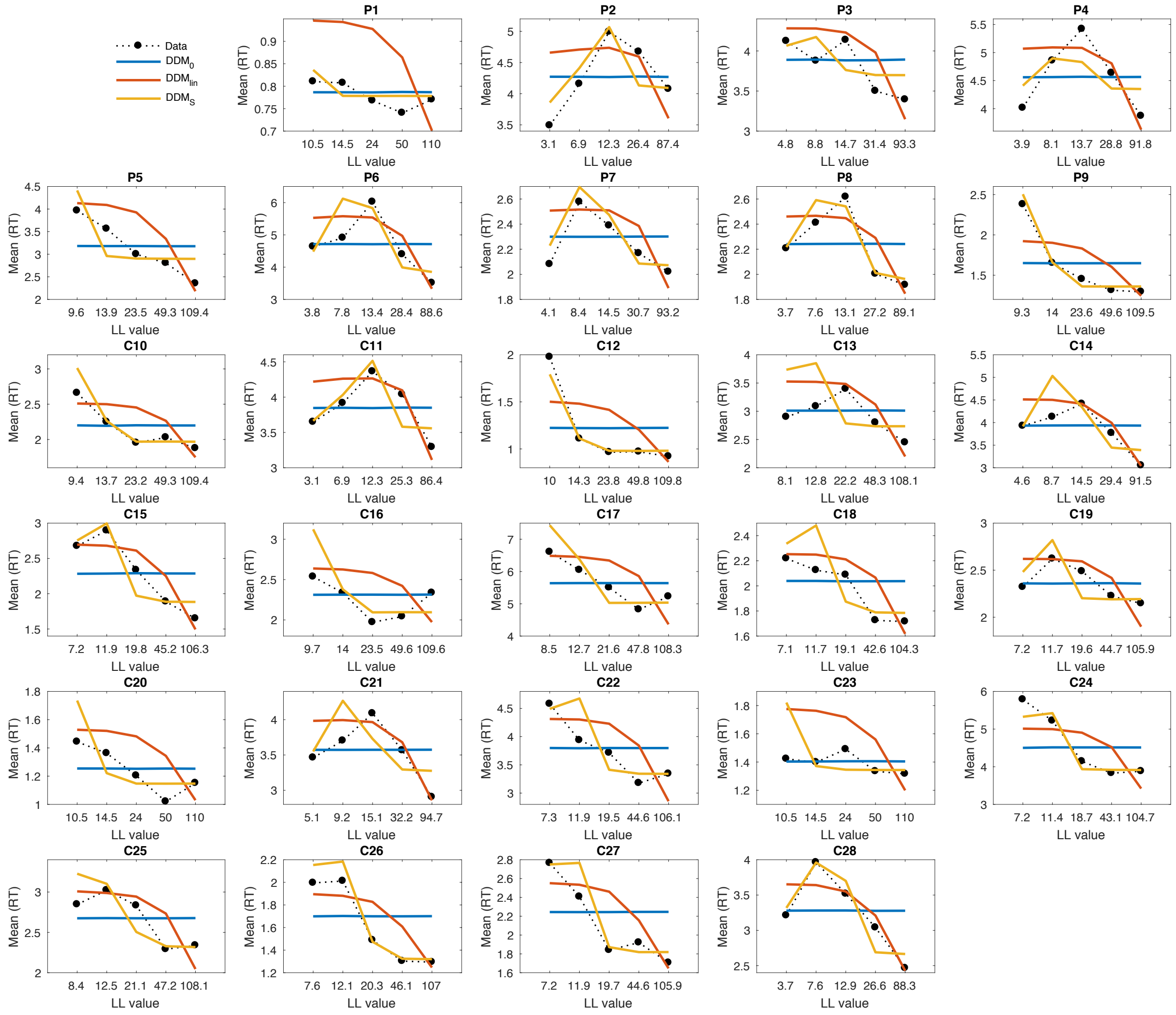

Figure 2. Posterior predictive plots for the different temporal discounting DDM models for all individual participants (P – mOFC patients, C – controls). Trials were binned into five bins of equal sizes according to the subjective value of the larger-later (LL) option for each participant (calculated according to equation 1). The x-axis in each panel shows the subject-specific mean LL value for each bin. The y-axis denotes observed response times per bin (dotted black lines) and model predicted response times per bin for the different DDM models (blue: DDM_0, red: DDM_{lin}, orange: DDM_S). Model predicted response times were obtained by averaging over 10k data sets simulated from the posterior distribution of each hierarchical model.

Figure 3 shows this for risky choice (see response to Reviewer #1 above). These improved posterior predictive checks show that the DDM_S provides a much better account of the relationship between RT and subjective value than the DDM_{lin}. We now expand on the likely reasons for this in the results section, where we now write on p.14 and p.16:

While the DDM_{lin} could account for some aspects of the association between values and response times in some participants, the DDM_S provided a much better account of this relationship overall.

This was in many cases due to the DDM_{lin} overestimating RTs (underestimating the drift rate) for intermediate value trials and underestimating RTs (overestimating the drift rate)

for trials with high value LL or risky options. This effect is most clearly seen in the temporal discounting data (Figure 2) where a greater proportion of value bins fall into the intermediate range. In the supplemental information, we visually compare predicted drift rates between DDM_{lin} and DDM_S to illustrate this effect (S6 Figure). Taken together, these analyses show that 1) the DDM_S provided an overall superior fit to both temporal discounting and risky choice data and 2) that this was reflected in a better account of both binary choices and the relationship between RTs and value.

We now also include an additional Figure S6 in the SI that illustrates the effect that the DDM_{lin} tends to overestimate RTs (underestimate the drift rate) for low value differences and underestimates RTs (overestimates the drift rate) when value differences are very large (see Figure S6 below). This effect can also be observed in a number of individual subject posterior predictive checks (see for example subject C17 in Figure 2 above).

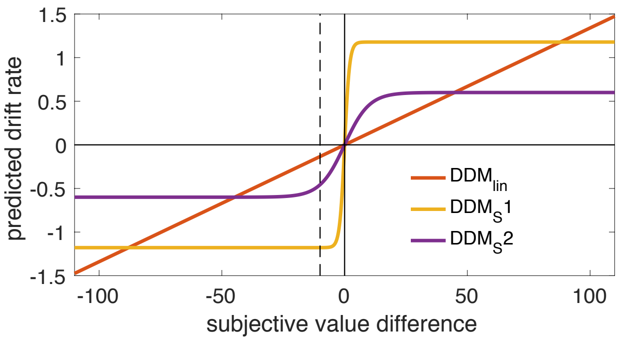

S6 Figure. Illustration of the differential effects of linear vs. sigmoid drift rate scaling. Linear scaling predicts longer RTs (lower drift rates) than sigmoid scaling for all but the greatest value differences, where the effect reverses. The reversal point depends on the drift rate components (DDM_S1: v_{max}=1.1786, v_{coeff}=.997, DDM_S2: v_{max}=.6, v_{coeff}=.2). The dashed line marks a value difference of -10, which was the lower bound of value differences in the present experimental design (i.e., the case when the risky or larger-later option was discounted to almost 0).

Reviewer #2: c. On the same point above, does vmax/vcoef estimates reflected differently in different aspects of the RT distribution (e.g., the tail/leading edge of the distribution)? Maybe the authors can also include a simulation where vmax/vcoef are mapped to aspects of the RT distribution (e.g., using ex-Gaussian fitting). This might then be used to show why DDMs actually fit better with RT data compared to DDMlin (hoping it does fit better to both, rather than to choice behavior only).

Response: Thanks, this is again a very helpful point. To address this issue, we performed simulations using the DDM_S. Here we show how the predicted RT distributions change as a function of v_{max} and v_{coeff} parameters by examining a range of parameter combinations. We then additionally show how these parameters affect the predicted relationship between subjective value and RT. The novel section in the results section on p. 9 reads as follows:

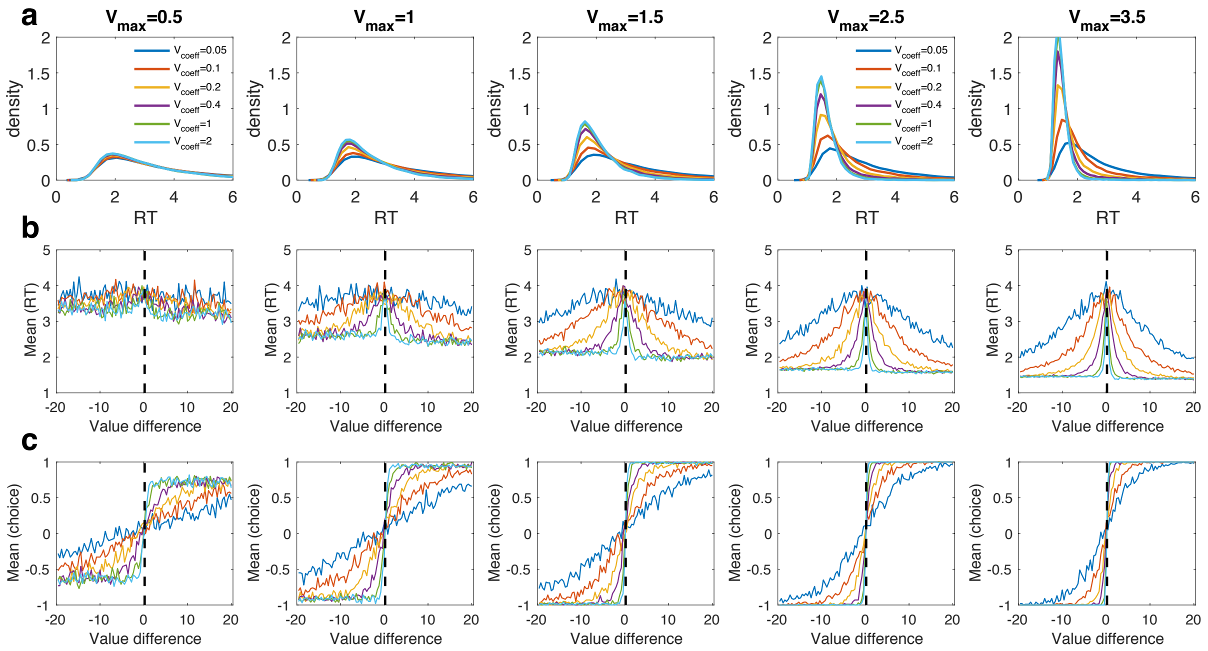

Figure 4. Simulation results for the DDM_S. a: Simulated RT distributions, b: Predicted mean RTs per value difference bin, c: predicted choice proportions per value difference bin. Simulation results are shown for a range of values of v_{max} (columns) and v_{coeff} (colored lines).

Simulations of effects of drift rate components on RT distributions

We next set out to more systematically explore how the two components of the drift rate in the DDM_S (v_{max} and v_{coeff}) affect RTs. To this end, we simulated 50 RTs from the DDM_S for each of 400 value differences ranging from zero to ± 20. We ran 30 simulations in total, systematically varying v_{max} and v_{coeff} while keeping the other DDM parameters (boundary separation, bias, non-decision time) fixed at mean posterior values of the control group (see Table 5).

Table 5. DDM parameter values used for simulation analyses depicted in Figure 4. All parameters are the posterior group means of the control group

	**Parameter value**
**Boundary separation** (α)	3.37
**Non decision time** (τ)	.945
**Starting point / bias** (z)	.531
**Drift rate *v* (max)**	[.5, 1, 1.5, 2.5, 3.5]
**Drift rate *v* (coeff)**	[.05, .1, .2, .4, 1, 2]

Simulated RT distributions are shown in Figure 4a, whereas mean simulated RTs and binary choices per value bin are shown in Figure 4b and 4c, respectively. Results from corresponding simulations computed across the actual delay/amount and probability/amount combinations from the tasks are shown in S8 (temporal discounting) and S9 (risky choice). As can be seen Figure 4a, the effects of v_{max} on the leading edge of the RT distribution were generally more pronounced for higher values of v_{coeff}. At the same time, smaller values of v_{coeff} generally lead to more heavy tailed RT distributions. The model of course predicts longest RTs for trials were values are most similar (the predicted RTs are highest for value differences close to zero, see the dotted lines in the right panels of Figure 4b). But the simulations illustrate an additional effect: Both relatively high and relatively low values of v_{coeff} can make RTs appear insensitive to value differences. For example, for the case of v_{coeff}=.05, RTs tend to be

uniformly slow, and accelerate only slightly for the largest value differences (blue lines in Figure 4b). In contrast, for the highest values of v_{coeff}, relatively small value differences already give rise to maximal drift rates and thus uniformly fast RTs for all but the smallest value differences (highest conflict).

We now also included a section elaborating on these simulation results in the discussion on p. 29, where we now write:

We also performed simulations to explore the impact of DDM_S drift rate components on the relationship between subjective value and RTs. These simulations revealed that both large and small values of v_{coeff} attenuate the non-linear relationship between value and RT: For very high values of v_{coeff} the DDM_S produces RT distributions with only marginally longer RTs for high conflict choices (green lines in Figure 4, this effect can also be seen in P1 in Figure 2, the participant with the highest v_{coeff} for temporal discounting of all participants). In contrast very low values of v_{coeff} yield RTs that tend to be uniformly longer for all but the easiest (highest value-difference) choices. The implication is that increases and decreases in v_{coeff} cannot unambiguously be interpreted as increases and decreases in value-sensitivity in RTs. Rather, as the simulations show, value-sensitivity (if interpreted as the degree of RT deceleration with increasing conflict) is maximal for intermediate values of v_{coeff}. At the same time, the magnitude of this effect depends on v_{max}.

Reviewer #2: d. What is the relationship between vcoef/vmax and discounting parameters? Does DDMs provide a better fit because it help to better capture the RT-SV associations – or rather help account for individuals where such a relationship is actually absent (e.g., maybe by allowing a high vcoef?)

Response: The issue of whether the DDM_S provides a better fit to the RT-SV association is addressed in the improved posterior predictive checks (see our extensive responses above). Regarding the association of the vcoeff/vmax and discounting parameters, we now included these analyses in the SI (S7 Figure and S2 Text), where we now write:

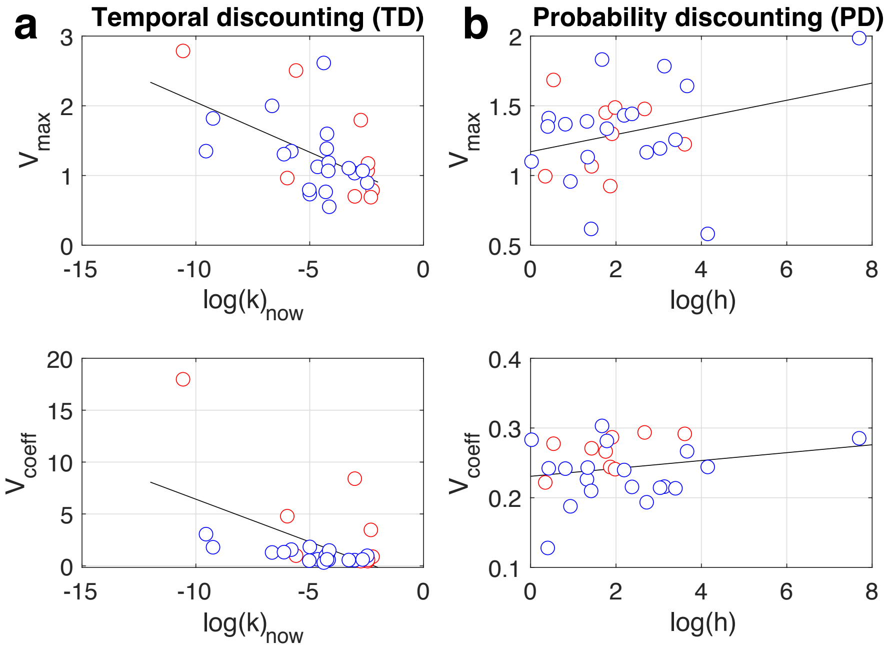

S7 Figure. Associations between drift rate components and discount rates for temporal discounting (a) and risky choice / probability discounting (b). Top panels show v_{max} and lower panels show v_{coeff}.

Drift rate components v_{max} and v_{coeff} and discounting behavior
We also explored the association between drift rate components and discounting behavior. S7 Figure plots the discount rates log(k) and log(h) against these parameters (means of individual subject posterior distributions for each parameter). Uncorrected non-parametric Spearman-correlation coefficients where as follows: $r_{log(k),\ vmax}$ =-.507, p=.0065; $r_{log(k),\ vcoeff}$ =-.432, p=.0227; $r_{log(h),\ vmax}$ =.216, p=.269; $r_{log(h),\ vcoeff}$ =.138, p=.482.

Reviewer #2: 2. The authors suggest two analysis which led them to the conclusion that DDM can be adequately used to describe value based decision (depicted in Fig4 and Fig5/6). However, I feel that these analyses are more of a 'sanity checks' rather than novel results:

a. The authors report high correlations between the same parameters fitted with either softmax or DDMs. Yet, since both models describe choice behavior – why would we expect the same parameter to differ due to the modeling of choice and RT combination vs choice only? I think this needs better justification/explanation. What did you have in mind? Did you expect RTs to change the model ability to accurately predict choice for some reason? I think the challenge here is not to show that DDM account for choices similar to softmax models (which means that modeling a combination of choice and RT as opposed to choice only, doesn't reduce the fit for choice data). The challenge here in my mind, is to show the benefits of modeling RTs and choices at the same time. Since modeling both RT and choice is more difficult, I think it should be justified by laying down the possible advantages of using such an approach.

b. Fig 5/6. Why would we expect anything else then a positive correlation between nd /th and min/median/(mean?) RT? I appreciate the fact that the authors are including this – but I don't see how this is more than a sanity check. Did you have any reason to believe that a value based DDM model will tamper with the nd/th relationship with RT? Why?
c. Further re "we re-ran the correlation only in the control group. Here, the correlation was lower but still robust (r=.52)." I would suggest this is very low. Why is that? Maybe it has to do with the recoverability of this parameter specifically, or maybe very low effect/variance between conditions for controls? The point is that this might be unrelated to the difference between DDMs and softmax models.

Response: This issue was also raised by Reviewer #1. In line with these comments, we have now moved all of these analyses into the SI and now clearly label them as "sanity checks" rather that novel results in the results section and discussion.

In the results section, we replaced the previous section (which is now included in the SI) with the following short section:

Model validation

We first carried out a number of simple sanity checks (see S1 Text) which confirmed that log(k) parameters estimated via standard softmax and via the DDM_s showed good correspondence (S3 Figure). Likewise, minimum and median RT showed the expected associations with model-based non-decision times (S4 Figure) and boundary separation parameters (S5 Figure).

And in the discussion, we now write:
We examined variants of the DDM in tasks where they have not been applied previously (although other sequential sampling models have[16]). We therefore ran a number of initial sanity checks to validate our modeling results (see SI) that generally supported the validity of our approach.

Reviewer #2: 3. Group differences look very interesting and valuable, but it's not clear whether you use these to validate the use of DDM, justifying the use of DDM for value based-decisions, or add to the mOFC literature per-se:

a. If group differences in nd is unrelated to value based processes – why is this a demonstration of why DDM can be beneficial here? Couldn't this be done with perceptual based decisions as well?

Response: This is an interesting point. One could speculate that non-decision times in perceptual decision-making might similarly be affected by vmPFC/mOFC damage, but we are not aware of a study explicitly testing this. Although it is well established that PFC contributes to perceptual decision-making, here theoretical accounts typically focus on DLPFC rather than ventro-medial regions. We now address this issue in the discussion, where we now write on p. 20:

In contrast, results from both tasks revealed a substantial increase in non-decision times in the patient group. Whether this effect is specific to value-based decisions or extends to other choice settings is an open question. However, accounts of perceptual decision-making have typically focused on lateral prefrontal cortex regions[62,63].

b. The authors report similar vmax, but higher vcoef for mOFC group. They conclude that "value-differences exert a similar (if not stronger) effect on trial-wise drift rates in vmPFC/mOFC patients compared to controls". I am not sure I understand why this led them to conclude that value choice processes are intact in mOFC patients. I think high vcoef might actually be a way of the model to account for participants that are insensitive to value differences.

Response: This is a good point. As can be seen from our simulations using the DDM_S (Figure 4 above), a high vcoeff parameter can in fact produce RT distributions that *appear* to be relatively insensitive to value differences, because only the smallest value-differences lead to an RT deceleration (see the green lines in Figure 4). For trials with larger value differences, trial-wise drift rates are rapidly mapped onto vmax, producing overall predominantly flat RT-value-difference graphs (with the notable exception of high conflict trials).

These effects can be illustrated by looking at the three participants with the highest vcoeff parameters. P1 for example had the highest vcoeff (17.89) for temporal discounting (see Figure 3 panel 1 from the improved posterior predictive plots), and shows a slight RT deceleration only for the highest conflict trials (which is captured by the DDM_S, but not the DDM_{lin}). In this particular case, the flat RT curve produced by the DDM_0 also constituted a relatively good fit to the data. However, this was not the case for P3 and P5, with the second and third highest vcoeff parameters (8.32 and 4.70). In both patients, RTs showed a clear modulation by value that was better accounted for by the DDM_S than both DDM_0 and DDM_{lin}. Note that what complicates the interpretation of the individual participant plots in Figure 3 and 4 is of course that these effects also depend on the discount rates and the option space

(amount and delay/probability combinations), and thus on the distribution of subjective values that a participant was exposed to during the task.

Reviewer #1 suggested a more direct test of value-sensitivity in the patient group using mixture models. That is, we examined whether groups differed on the proportion of trials that were best accounted for by the DDM_S as opposed to the null model DDM_0. The mixture models contained full hierarchical parameter sets of both models as well as a mixture parameter that modeled the proportion of trials produced by the DDM_0. The vast majority of trials was better accounted for by the DDM_S than the DDM_0 for both tasks with little evidence for a group difference ($.5 < BF < 2.1$):

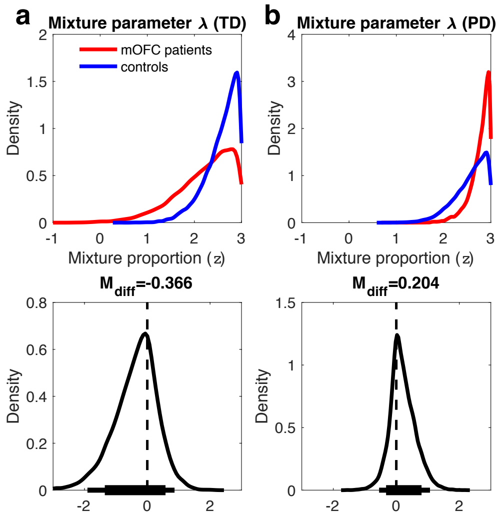

Figure 9. Top row: posterior distributions of the mixture parameter λ (a: temporal discounting (TD), b: risky choice / probability discounting (PD)) in z-units. Positive values of λ indicate that a greater proportion of trials was better accounted for by DDM_S vs. DDM_0, whereas negative values indicate the reverse. λ was fitted in standard normal space with a group-level uniform prior of [-3, 3] and backtransformed on the subject-level via an inverse probit transformation. Bottom row: Posterior group differences (mOFC patients – controls) for each parameter. Solid horizontal lines indicate highest density intervals (HDI, thick lines: 85% HDI, thin lines: 95% HDI).

Reviewer #2: c. Is it possible that the difference in starting point is only due to choice data, but not RT?

Response: Yes, we cannot rule this out, and now explicitly mention this in the discussion of bias effects on p. 19 of the discussion, where we now state:

That is, the group that displayed a preference for one option as reflected in the discount rate parameter (e.g. LL rewards in the case of controls) also exhibited a response bias towards that decision boundary. It should be noted that these numerical differences in bias could be attributable to differences in the RT distributions, differences in the binary choices, or both.

Reviewer #2: 4. The issue of accuracy vs. stimulus coding is emphasized. However, it was hard for me to follow why this makes a difference. The assignment of the upper boundary to the higher value option is, to the best of my understating, strictly technical (both models are perfectly equivalent). I think it's good to note the differences, but I'm not sure I understand why it emphasized (i.e., had a paragraph both in the intro and discussion).

Response: In line with these suggestions, we have removed the extensive section on stimulus vs. accuracy coding from the introduction, and shortened the section in the discussion. However, we still retained parts of the discussion section, since we believe that it is important to mention the implications of the coding scheme for parameter interpretation and for the possibility to estimate a bias parameter.
The corresponding section on p. 18/19 of the discussion now reads:

The stimulus coding scheme (coding the boundaries in terms LL/risky options vs. SS/safe options) that we adopted here differs from accuracy coding as implemented in recent applications of the DDM to reinforcement learning[14,15] (coding the boundaries in terms of correct vs. incorrect choices), with implications for the interpretation of the DDM parameters. The drift rate v in the present coding scheme (as reflected in v_{max} and v_{coeff}) can be interpreted as in classical perceptual decision-making tasks: it reflects the rate of evidence accumulation. In stimulus coding, however, higher drift rates do not directly correspond to better performance (as is the case in accuracy coding), because there is no objectively correct response. Instead the drift rate parameters reflect a participant's overall sensitivity to value differences, similar to inverse temperature parameters in softmax models. More importantly, adopting stimulus coding allowed us to estimate a starting point (bias) parameter. In all cases, the estimated bias parameters were relatively close to 0.5 (a neutral bias), but group differences for each task mirrored the results for the choice model parameters. That is, the group that displayed a preference for one option as reflected in the discount rate parameter (e.g. LL rewards in the case of controls) also exhibited a response bias towards that decision boundary. It should be noted that these numerical differences in bias could be attributable to differences in the RT distributions, differences in the binary choices, or both.
