# Supplementary material for: The drift diffusion model as the choice rule in inter-temporal and risky choice: A case study in medial orbitofrontal cortex lesion patients and controls
Source: PLoS Comput Biol. 2020 Apr 20;16(4):e1007615. doi: 10.1371/journal.pcbi.1007615 (PMC7192518; doi:10.1371/journal.pcbi.1007615)
Supplement: Attachment — Submitted filename: Peters_ResponseLetterR2.pdf [file pcbi.1007615.s014.pdf]

Peters & D'Esposito. The drift diffusion model as the choice rule in inter-temporal and risky decision-making (R2).

Response to reviewer's comments

Reviewer #1: Thank you for the additional mixed-models analyses. I only have one question about that, and it's why was the lambda parameter constrained to be [-3, 3] in the standard normal space. Judging by the posteriors, it looks like the distributions are all pushing towards the bound at 3. If possible, I would consider relaxing the prior distribution to accommodate higher values of lambda.

Response: Thanks for pointing this out. We re-ran the analysis with a relaxed prior distribution of [-7, 7] in standard normal space (see new Figure 9 below) and this revealed the same result. However, we still observed some posteriors pushing towards the bounds. This reflects the fact that the DDMs provides a better account of on average the vast majority of trials – as the proportion of trials better accounted for by the DDMs goes towards 1, lambda in z-space will go towards +Inf, so relaxing the prior even more would provide no additional information. We now also emphasize that the group differences in lambda when transformed to raw proportion units are minuscule, and state in the respective section in the results section (p.16):

"Thus, on average, in both groups >>99% of trials were better accounted for by the DDM_S compared to the DDM_0. Because group differences in lambda are minuscule in raw proportion units, they were not further examined."

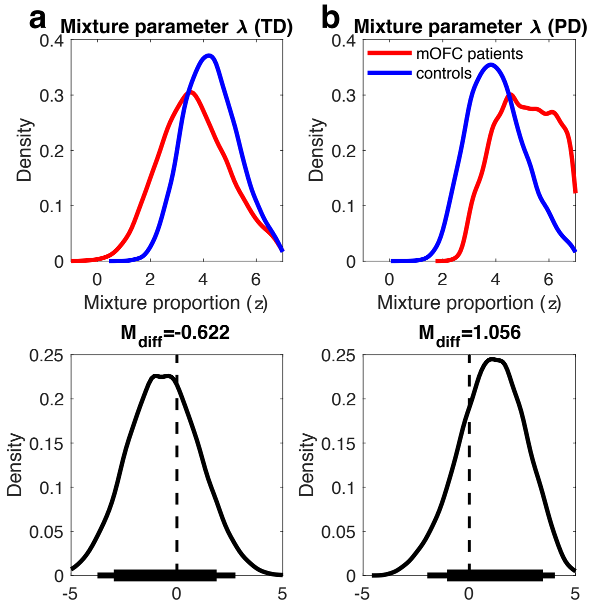

Figure 9. Top row: posterior distributions of the mixture parameter λ (a: temporal discounting (TD), b: risky choice / probability discounting (PD)) in z-units. Positive values of λ indicate that a greater proportion of trials was better accounted for by DDM_S vs. DDM_0, whereas negative values indicate the reverse. λ was fitted in standard normal space with a group-level uniform prior of [-7, 7] and backtransformed on the subject-level via an inverse probit transformation. Bottom row: Posterior group differences (mOFC patients – controls)

for each parameter. Solid horizontal lines indicate highest density intervals (HDI, thick lines: 85% HDI, thin lines: 95% HDI).
